# Pregnancy Outcome Following First-Trimester Exposure to Bilastine: A Comparative Observational Cohort Study from the Israeli Teratology Information Service

**DOI:** 10.3390/jcm15135035

**Published:** 2026-06-28

**Authors:** Reem Hegla-Murad, Svetlana Shechtman, Orna Diav-Citrin

**Affiliations:** 1 The Israeli Teratology Information Service, Ministry of Health, Jerusalem 9446724, Israel; reem.murad@moh.gov.il (R.H.-M.); svetlana.shechtman@moh.gov.il (S.S.); 2 Department of Medical Neurobiology, The Hebrew University and Hadassah Medical School, Jerusalem 9112102, Israel

**Keywords:** bilastine, antihistamines, pregnancy, major anomalies

## Abstract

**Background**: Bilastine is a long-acting, nonsedating antihistamine used to treat allergic conditions. Human pregnancy experience with bilastine remains limited. **Objectives**: The primary aim was to evaluate the risk of major anomalies following first-trimester bilastine exposure. Secondary endpoints included additional pregnancy outcomes. **Methods**: This observational, prospective cohort study included women counseled by the Israeli Teratology Information Service regarding first-trimester bilastine exposure between July 2019 and June 2023. Participants were contacted by telephone for follow-up using a structured questionnaire. Pregnancy outcomes were compared with fexofenadine-exposed pregnancies. The data analysis, comparison, and presentation followed the recommendations of the Strengthening the Reporting of Observational Studies in Epidemiology (STROBE) guide. **Results**: Follow-up was obtained for 36 pregnancies with at least first-trimester bilastine exposure. The median daily bilastine dose was 20 mg, with treatment discontinued at a median gestational age of 5 + 6 weeks. First-trimester bilastine exposure was not associated with an increased risk of major anomalies [bilastine: 2/33, 6.1% vs. fexofenadine: 2/27, 7.4%; crude OR 0.81, 95% CI 0.11–6.14]. No pattern of malformations was observed. Rates of live-birth, miscarriage, and pregnancy termination were 91.7%, 5.6%, and 2.8% in the bilastine group, and 72.2%, 19.4%, and 8.3% in the fexofenadine group, respectively. **Conclusions**: This cohort study, although small, provides preliminary reassurance for counseling regarding inadvertent early pregnancy exposure to bilastine. Larger studies are needed to validate these findings.

## 1. Introduction

The use of antihistamines during pregnancy has evolved significantly over the past decades, with a market shift from first-generation to second-generation agents. The use of antihistamines during pregnancy is prevalent. Second-generation antihistamines, including loratadine, cetirizine, and fexofenadine, have become common. The introduction of newer agents like bilastine represents the latest advancement in this therapeutic class. However, prescribing patterns vary significantly across different health care systems, with some regions showing conservative approaches to newer antihistamines.

Bilastine is a novel, second-generation piperidine derivative [1] that acts as a highly selective, long-acting peripheral histamine H1-receptor antagonist and inverse agonist [2]. It binds competitively to the H1-receptor, preventing histamine binding and stabilizing the receptor in its inactive conformation, thereby reducing basal receptor signaling activity. Beyond receptor blockade, bilastine also exhibits anti-inflammatory properties by inhibiting the release of key pro-inflammatory mediators [3]. It also suppresses eosinophil migration to the inflamed tissues, thereby stabilizing mast cell membranes [3].

Bilastine is used in the symptomatic treatment of allergic conditions, such as allergic rhinoconjunctivitis and urticaria [4]. Its clinical utility stems from favorable pharmacokinetic properties, including rapid onset and long duration of action. The drug’s potency is based on its receptor affinity profile, exceeding that of other widely utilized second-generation antihistamines [5]. Bilastine does not readily cross the blood–brain barrier. It, therefore, remains nonsedating, even at supra-therapeutic doses, and does not impair cognitive and motor performance. It undergoes minimal hepatic metabolism and therefore does not interact with the cytochrome P450 enzyme system, offering an advantage in managing patients on multi-drug regimens [1]. Another distinguishing feature of bilastine is its receptor selectivity, which prevents the anticholinergic side effects common to first-generation antihistamines [3]. The drug’s bioavailability is highly sensitive to food intake, requiring administration on an empty stomach. Food and fruit juices, particularly grapefruit juice, can result in reduced systemic exposure of bilastine. As a regulatory precaution, bilastine should not be given to patients with a history of QT prolongation or severe arrhythmias. In addition, it should not be co-administered with P-glycoprotein inhibitors, such as verapamil, quinidine, erythromycin, clarithromycin, ketoconazole, itraconazole, and cyclosporine, in patients with moderate-to-severe renal impairment [6].

In the preclinical animal reproductive studies, performed in rats and rabbits, bilastine administered at doses of up to 1000 mg/kg/day and up to 400 mg/kg/day, respectively, during the period of major organogenesis did not show a teratogenic effect [7]. Bilastine was well tolerated in pregnant and lactating rats, as well as in their offspring and subsequent generations [7]. Human pregnancy experience with bilastine is scarce. According to its product monograph, 12 pregnancies were reported in women exposed to bilastine; all pregnancies had normal outcomes, except for one pregnancy termination in a woman with a history of recurrent miscarriages and antiphospholipid syndrome [6]. Treating allergic conditions during pregnancy is a significant clinical challenge. Untreated allergic conditions can significantly influence maternal quality of life, on the one hand, while potential effects on pregnancy outcome might pose embryofetal and maternal risks, on the other hand. Allergic rhinoconjunctivitis and urticaria are common in childbearing age. The median worldwide prevalence of allergic rhinitis is 18.1% in adults [8]. Both conditions predominantly affect women [9,10]. In an international multicenter study on the course of chronic urticaria during pregnancy, it improved in 51.1% of patients, worsened in 28.9%, and was unchanged in 20.0%, with exacerbations most commonly reported exclusively during the third (27.6%) or the first (22.8%) trimesters [11]. To date, there are no adequate and well-controlled studies in pregnant women on bilastine. Fexofenadine is a second-generation antihistamine with more human data on its safe use in pregnancy [12].

Driven by a transition away from older antihistamines, prescriber preference has shifted toward using the generally safe and highly effective bilastine. Despite its favorable preclinical data, there is a paucity of human pregnancy clinical experience. With increasing bilastine use, inadvertent exposure during pregnancy is anticipated. The aim of the present study was to assess pregnancy outcomes following at least first-trimester exposure to bilastine compared with fexofenadine-exposed pregnancies. The primary objective was to evaluate the risk of major anomalies. Secondary endpoints included live-birth, miscarriage, and elective termination of pregnancy, gestational age at delivery, preterm delivery (birth before 37 completed weeks), and birth weight.

## 2. Materials and Methods

The study is a single-center, prospective, observational cohort study. The Israeli Teratology Information Service (TIS) provides individual counseling services with regard to environmental exposures during pregnancy and lactation, including medications, infections, exposure to ionizing radiation, and maternal diseases. TIS providers offer risk assessment to pregnant women who contact the service. Methodological aspects pertaining to TIS studies of a similar nature have been extensively documented in the existing literature, thus serving as a reference for performing the present study [13,14,15].

The TIS database was searched for contacts regarding bilastine during pregnancy from July 2019 until June 2023. Women counseled by the TIS about bilastine during the first trimester of pregnancy were contacted by telephone for follow-up using a structured questionnaire. Pregnancy exposure was prospectively ascertained at initial contact, before pregnancy outcome was known, and before any major anomalies were detected. The following information was recorded at the initial contact: maternal characteristics, such as age, tobacco use, and alcohol consumption, and medical and obstetric history, as well as exposure details (dose, duration, timing in pregnancy, indication, additional exposures).

Follow-up was performed via telephone interview after the expected date of delivery. Interviews were conducted by trained personnel who captured detailed information about pregnancy outcome, gestational age at delivery, birth weight, and presence of congenital anomalies. In addition, all exposures were ascertained. Multiple contact attempts were made to maximize response rate. No review of medical charts was performed.

Pregnancy outcome was compared with fexofenadine-exposed pregnancies during the first trimester from the TIS database in similar years at a 1:1 ratio. Pregnancies in the comparison group were randomly selected.

In both groups, only participants with prospectively ascertained pregnancy outcomes, meaning those with unknown pregnancy outcomes or prenatal diagnosis at the time of study enrollment, were included. Both groups had no defined limit on the duration of exposure.

Pregnancies with exposure to teratogenic drugs (such as systemic retinoids, including acitretin, alitretinoin, isotretinoin; cytotoxic agents; selected antiepileptic drugs including valproate, carbamazepine, phenytoin, topiramate, phenobarbital; thalidomide; coumarin derivatives such as warfarin and acenocoumarol; lithium; misoprostol; methimazole) at any time during pregnancy were excluded. In addition, exposure to angiotensin converting enzyme inhibitors, angiotensin II receptor blockers, or tetracyclines during the second and/or third trimesters, presence of malignancies or malignancy-related conditions were also excluded. Duplicate cases were also excluded.

The primary outcome of interest was non-chromosomal major anomalies. Each neonate underwent at least two physical examinations before discharge from hospital. Major anomalies were defined as structural non-chromosomal abnormalities in offspring that have serious medical, surgical, or cosmetic consequences. Congenital anomalies were classified by a certified pediatrician (ODC) blinded to the exposure group. The classification followed the European Network of population-based registries for the epidemiological surveillance of congenital anomalies (EUROCAT) guideline [16]. Ventricular septal defects were considered major anomalies in the current study. Secondary endpoints included the risks of spontaneous pregnancy loss; elective termination of pregnancy; neonatal birthweight; mode of delivery, which included uncomplicated vaginal delivery, Cesarean section, or assisted vacuum delivery; and gestational age at delivery. Gestational age was defined from the first day of the last menstrual period. The period of organogenesis was defined from gestational week 4 + 0 until week 12 + 6. Pregnant women exposed during the “all or none” period (the first two post-conception weeks) only, and women with multifetal pregnancies, were excluded from the analysis.

The data analysis, comparison, and presentation followed the recommendations of the Strengthening the Reporting of Observational Studies in Epidemiology (STROBE) guide [17]. Crude risk of major anomalies was determined by dividing the total number of infants or fetuses with non-chromosomal anomalies by the sum of all live-born infants and terminated pregnancies with known non-chromosomal anomalies.

Since the study is non-interventional, it did not require ethics approval by an Institutional Review Board.

The statistical analysis was done using IBM SPSS Statistics for windows software 30.0.0.0 (172) version IBM Corp. (2024), Armonk, NY, USA.

## 3. Results

Thirty-six bilastine-exposed pregnancies, during at least the period of organogenesis, were included in the study, and compared with 36 fexofenadine-exposed pregnancies. A comparison of maternal characteristics and obstetric history of the two groups is presented in Table 1. No significant differences were observed between the groups in maternal age, gestational age at initial contact with the TIS, pregnancy number, and history of miscarriage or elective termination of pregnancy.

The median daily bilastine dose was 20 mg. Treatment was discontinued at a median gestational age of 5 + 6 weeks. The women in our cohort did not smoke tobacco or consume alcohol during pregnancy. A comparison of pregnancy outcomes between the two groups is presented in Table 2. There were no significant differences in pregnancy outcome, neonatal birthweight, or mode of delivery.

First-trimester bilastine exposure was not associated with an increased risk of major anomalies [bilastine: 2/33, 6.1% vs. fexofenadine: 2/27, 7.4%; crude OR 0.81, 95% CI 0.11–6.14].

Major anomalies were reported in two bilastine-exposed offspring. A term male infant, whose mother has been treated with bilastine throughout pregnancy, had been diagnosed with pyloric stenosis and underwent corrective surgery at two months of age. The second was a female infant born with a ventricular septal defect (VSD), diagnosed in the neonatal period, and followed up by a pediatric cardiologist with serial bimonthly echocardiography exams planned until closure or one year of age.

In the fexofenadine-exposed group, there was an early pregnancy termination at gestational week 20 after prenatal diagnosis of hypoplastic left heart syndrome, and a male infant diagnosed at birth with a VSD that spontaneously closed by 8 months of age.

Significant differences were not observed in pregnancy outcomes. The rates of live-birth, miscarriage, and pregnancy termination were 91.7%, 5.6%, and 2.8% in the bilastine group, and 72.2%, 19.4%, and 8.3% in the fexofenadine group, respectively.

There were no preterm infants in the two groups.

## 4. Discussion

Antihistamines are commonly used medications during pregnancy for treating allergic rhinoconjunctivitis, urticaria, and nausea. The literature on the safety of antihistamine use during pregnancy with respect to congenital anomalies is generally reassuring [18]. First-generation H1-receptor antagonists can cross the blood–brain barrier with resulting sedative and anticholinergic side effects. Second-generation H1-receptor antagonists lack those effects. Among 31 cohort studies included in this systematic review [18], one study based on the Swedish Medical Birth Registry study identified a statistically significant positive association between loratadine and hypospadias [19]. Five years later, the same authors published a follow-up study and proposed that the 2001 “signal” had been a chance finding [20]. Loratadine has been thoroughly investigated with several TIS and case–control studies that have not shown an association between its use in pregnancy and congenital anomalies.

Bilastine is a potent second-generation, long-acting, nonsedating, selective H1-antihistamine indicated for treating allergic conditions such as allergic rhinoconjunctivitis and urticaria, both prevalent among women of reproductive age. These medical conditions are female predominant and adversely affect patients’ quality of life, sleep, and daily activities [21,22]. They often exacerbate during pregnancy. Patients require a fast-acting, effective, and nonsedating treatment. Bilastine meets these criteria, and its once-daily regimen offers improved compliance [23]. Bilastine was first approved in European countries and later by Health Canada and by the Australian Therapeutic Goods Administration. While bilastine is approved in over 120 countries, it remains unavailable in the United States. Bilastine is classified as Category B for pregnancy in most jurisdictions where it is approved, indicating that animal reproductive studies have not demonstrated embryofetal risk, but adequate human studies are lacking. Animal reproduction studies did not show a teratogenic signal, while human pregnancy experience with bilastine is very limited. In the present study, bilastine exposure during organogenesis was not associated with an increased risk of major anomalies compared with fexofenadine prenatally exposed offspring. The reported malformations in the bilastine group involve different organ systems and do not suggest a pattern. The VSDs reported in our study did not require any intervention, and one spontaneously closed during the study period.

To the best of our knowledge, the present study is the first comparative cohort on bilastine-exposed pregnancies. Exposure ascertainment preceded obtaining the pregnancy outcome. Data were available on medication dose and timing of exposure in pregnancy, allowing assessment of exposure during the period critical for organ formation, excluding exposure in the “all or none” period. The study includes data on pregnancy losses. The classification of major anomalies was performed blinded to the exposure. The comparison group was collected from the same TIS database and exposed to fexofenadine, an antihistamine with more data on its safe use in pregnancy, often used for similar indications. This design has high internal validity and minimizes potential recall and other biases. The primary limitation of this study is its small sample size, which limits its statistical power in general and for rare anomalies. Additional limitations include insufficient data on full first-trimester exposure, as well as on continuous or later-trimester bilastine exposure, potential residual recall bias, lack of adjustment for confounding variables, and absence of anomaly data among pregnancies resulting in a miscarriage or in an early elective termination.

Future research priorities should focus on expanding the evidence base through international collaborative efforts. Large-scale pregnancy registries, similar to those established for other medications, could provide the statistical power necessary to detect teratogenic risks or provide safety data. Studies are required to assess pregnancy outcome and potential neonatal effects after ongoing bilastine treatment, or during the second or third trimesters, and to evaluate long-term neurodevelopmental milestones and growth outcomes in children of mothers who took bilastine during pregnancy. Additionally, comparative effectiveness research evaluating bilastine against established antihistamines in pregnant populations could help establish evidence-based treatment guidelines. In vivo studies looking into the mechanistic insights on potential pregnancy and neonatal effects of bilastine might also be helpful.

## 5. Conclusions

The present prospective cohort study represents an important initial step in offering preliminary reassurance for counseling women in case of inadvertent early pregnancy exposure to bilastine, while the small sample size precludes definitive conclusions about its safety profile in pregnancy. These findings support the continued development of larger studies to validate and expand these initial observations. We hope that our study will prompt more research on bilastine during pregnancy.

## Figures and Tables

**Table 1:** Maternal characteristics and obstetric history.

Characteristic	Bilastine (n = 36)	Fexofenadine (n = 36)
Maternal age (y), median (IQR)	31 (28–35)	31 (29–34)
GA at initial contact (week), median (IQR)	6 (5–8)	7 (5–10)
Pregnancy number, n (%)		
1	17 (25.8)	18 (27.3)
2–4	15 (22.7)	13 (19.7)
≥5	2 (3.0)	1 (1.5)
Previous miscarriage, n (%)		
0	30 (41.7)	27 (37.5)
≥1	6 (8.3)	9 (12.5)
Previous ETOP, n (%)		
0	32 (88.9)	35 (97.2)
≥1	4 (11.1)	1 (2.8)

y = year, IQR interquartile range, GA = gestational age, ETOP = elective termination of pregnancy.

**Table 2:** Pregnancy outcomes and neonatal characteristics.

Outcome	Bilastine (n = 36)	Fexofenadine (n = 36)
Delivery of a live-born infant, n (%)	33 (91.7)	26 (72.2)
Miscarriage, n (%)	2 (5.6)	7 (19.4)
ETOP, n (%)	1 (2.8)	3 (8.3)
Gestational age at delivery, mean ± SD	39 ± 1	40 ± 1
Birthweight (grams), median (IQR)	3113 (2981–3589)	3375 (3119–3658)
Mode of delivery:		
Uncomplicated vaginal, n (%)	23 (69.7)	12 (50.0)
C/S, n (%)	6 (18.2)	6 (25.0)
Vacuum, n (%)	4 (12.1)	6 (25.0)
Non-chromosomal major anomalies *	2/33 (6.1)	2 **/27 ** (7.4)

ETOP = elective termination of pregnancy, C/S = Cesarean section, * including anomalies among live-births or in ETOPs, ** one ETOP due to anomalies included.

## Data Availability

The original contributions presented in this study are included in the article. Further inquiries can be directed to the corresponding author(s).

## Abbreviations

The following abbreviations are used in this manuscript:

TIS	Teratology Information Service
VSD	Ventricular Septal Defect
